# Core Competencies for Health Workers to Deal with Climate and Environmental Change

**DOI:** 10.3390/ijerph18083849

**Published:** 2021-04-07

**Authors:** Paul Jagals, Kristie Ebi

**Affiliations:** 1 Children’s Health and Environment Program, University of Queensland, South Brisbane, QLD 4102, Australia; 2 Departments of Global Health and of Environmental and Occupational Health Sciences, University of Washington, Seattle, WA 98199, USA; krisebi@uw.edu

**Keywords:** health workforce, core competencies, climate change, environmental change, HESET, environmental-public health

## Abstract

Rapid, detrimental climate change and environmental degradation pose real threats to the health, environment, social, economic and technological wellbeing of society (HESET). It has become even more imperative that the health workforce (public health and medical healthcare as well as auxiliary and support workers) be ‘climate-environment’ competent to fulfil their role in managing the environmental public health risks and impacts as climate and environment inevitably continue to change. We developed a broad six-domain competency framework consisting of (1) climate and environment sciences, (2) drivers of climate change (3) evidence, projections and assessments (4) iterative risk management (5) mitigation, adaptation and health co-benefits and (6) collective strategies—harnessing international/regional/local agreements and frameworks. The framework can be used by health/medical trainers to design cross-sectoral sub-competencies and learning content for training health workers to function at local, regional and global levels. Reaching, maintaining and improving the different levels of competency, the health workforce will be increasingly invaluable partners in intra- as well as inter-sectoral responses to climate and environmental risks and impacts.

## 1. Introduction

Rapidly changing anthropogenically-induced climate and other significant environmental changes pose serious threats to human health and well-being by exacerbating existing health inequities, disproportionately affecting vulnerable populations, detrimentally altering the social and environmental determinants of health, and by damaging health-supporting infrastructure [1,2,3,4]. These environmental and climate-related challenges pose real threats to society at local, regional and global levels and are among the key priorities for WHO [2], UNEP [5], UNDP [6], UNICEF [7] to prepare for—and protect against—environmental health emergencies, as well as promote sustainable and resilient development across all the health, environment, social, economic and technological (HESET) wellbeing domains of society.

For health services to remain consistently safe and operational during extreme/disastrous events and conditions-natural and anthropogenic, will require substantial and increased investment and effort, not just in physical infrastructure but especially in increasing competence of health workers. As climate and environmental change are outside the traditional training and continual professional development of the health workforce, few have the knowledge and skills to effectively prepare for, and manage extreme weather and climate events, infectious disease outbreaks, hazardous industrial releases and more [5,8].

Current and next generation health workers (public and medical health as well as auxiliary and support) should be suitably capacitated to prepare for, and appropriately respond to risks and impacts from climate and environmental change, especially health workers at the forefront of delivering public, preventive, and curative health services [9].

This article proposes a broad set of core competencies aimed to augment existing health worker competencies that range from clinical-focused roles to community and public health-focused, including more specialized roles such as environmental health workers and public health nurses and doctors. Competencies are the observable abilities of a person to perform tasks (or practices). Competencies are demonstrated through actions (behaviours) that integrate knowledge and skills to the levels that their specific responsibilities require. Put in another way, within the workforce, the efficiency of work-at the required level of such work-reflects competence [10,11,12].

In the context of climate and other environmental changes, we focus on environmental and public health roles, although health workers in clinical roles should also be competent in some-if not all-of the aspects described here, depending on their specific roles (i.e., preparing hospitals for a pending/projected natural disaster). The set of core competencies we propose is suitably flexible to capacitate climate, environmental change, and health (CECH) competence, as well as competence for clinical health workers.

All health workers who are competent in CECH should know and understand the ways that climate, environment, and health intersect. To contribute towards preparation, response, and recovery efforts in adverse climate- and environmentally related events, health workers also need to know and understand how CECH risks and impacts are managed–from local to global.

Such a set of CECH competencies should thus be rooted in the collective understanding of climate, environmental, and associated health sciences and the upstream drivers of these; effective intervention strategies based on mitigation and adaptation; and how health service demands may vary in concert with changes in climate and our diverse environments. Depending on their responsibilities as determined by their practices and professions, CECH-competent health workers might be required–to varying extents-to apply such knowledge and understanding when collaborating also with other disciplines in the design, implementation, and monitoring of effective interventions.

The health sector, through its local, country, regional and global levels of health governance, needs to be an effective partner with other government, community, private and academic sectors on CECH issues [1,9,12,13,14]. To develop sectoral partnerships to manage CECH, health workers should therefore also be competent in knowing and understanding the roles of-and interactions with–these other sectors [13]. Such partnerships not only will encourage the other sectors to include health considerations into their own climate and environment policies and actions–these other sectors could also then support the health sector in monitoring, preventing and reducing health risks and impacts. Partnerships will help the health sector to minimize its own climate and environmental footprint (caused by health-supporting systems and infrastructure), and in turn lend support to other sectors by advocating for and advising on measures and interventions to address all forms and sources of environmental impact such as pollution and degradation [1,2,5].

In this complex context, the competencies proposed in this article were developed from review and understanding of key climate and environment competencies for health workers in science and governance literature and frameworks-and how these should be defined and framed to prepare health workers to address, prepare for, and act on current and future health risks and impacts of climate and environmental change.

We systematically searched-and then conducted a narrative review-of current and past literature and other sources of information, i.e., university and governance websites [15,16]. We specifically screened for information about health worker competencies as well as for other relevant competency approaches associated with CECH, using keywords that underpin climate change, environmental change and health.

For formal higher education institutions that offer (or are planning to offer) training in health sector-based climate and environmental public health science, practice, and management, this article provides a CECH framework upon which to base more detailed development of sub-competencies. Furthermore, there is also a need to increase CECH competency development within the current health and broader workforce–this framework could guide strategies and policies of ministries of health and other ministries to achieve this. The framework is proposed to be a simple domain-based guide to trainers about the core CECH competencies that should be considered for university and other training institutions curricula used to educate and train new graduate members of the health workforce as well as continuing professional development of current health workers.

## 2. Revisiting the Concepts

**Figure 1:**
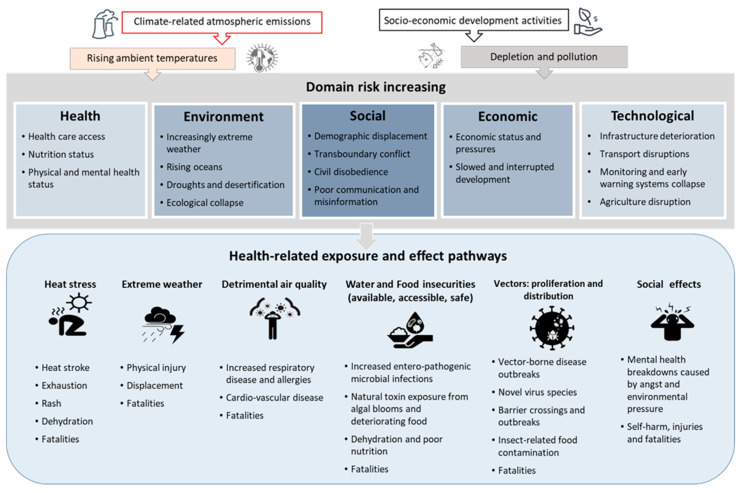
Examples of major health risks associated with climate and environmental change. This Figure 1 is developed from insights from [3,5,17] and is not intended to be comprehensive.

To provide a base for the core CECH competencies, we provide a brief and simplified review of some of the major concepts of ‘Climate’ and ‘Environmental’ Change in the context of ‘Health’ (Figure 1) and then consolidate them in the knowledge and application competencies summarised in Table 1 (Section 4).

### 2.1. Climate Change and Health

Climate change is perhaps the most extensively reported of the global environmental changes affecting human health and well-being [5]. Climate change acts through multiple pathways [4,18,19]:

- Changes in the frequency and intensity of extreme weather and climate events, including heat, windstorms, flooding, and drought;
- Effects mediated through ecological and environmental systems for example changes in the geographic range and incidence of infectious diseases (water-, food-, and vector-borne diseases), and poor air quality (excessive concentrations of ozone, particulate matter, dust, aeroallergens); and
- Effects heavily mediated by human systems (e.g., urbanisation, occupational impacts, undernutrition, conflict, population growth and migration, and mental stress).

Cascading and compounding effects from climate-related shocks and stresses can directly disrupt access to health and social services, affecting the quality and comprehensiveness of health care when, for example, infrastructure is damaged, or supply chains are disrupted.

Estimates of the overall health burden of climate change are almost certainly underestimates because of the complexity of the causal pathways between climate-related hazards and climate-sensitive health outcomes and a lack of robust surveillance and monitoring systems in many countries [4]. As climate continues to change over the century, along with population health status and health system capacity, thresholds can be crossed that could result in large fluctuations in the incidence of climate-sensitive health outcomes [20].

### 2.2. Environmental Change and Health

Other environmental changes can be caused by changing climate–but also by independent factors driven by human need for development and economic growth. Such activities lead to degrading ecological capital change such as land-system change, increased freshwater overuse, disrupted biochemical flows (e.g., excessive phosphorus and nitrogen discharges), ocean acidification, detrimental atmospheric aerosol and particle loading, stratospheric ozone depletion, and biodiversity loss–all which will have indirect and indirect effects on health [21,22]. For instance stratospheric ozone depletion can increase skin cancer, deforestation can increase the rates of malaria, biodiversity loss reduces access to natural medicines, and unplanned settlements increase waterborne diseases from a lack of access to safe water and also inadequate sanitation [23].

### 2.3. The Health Response should Be Collective Across All Sectors of Society

**Figure 2:**
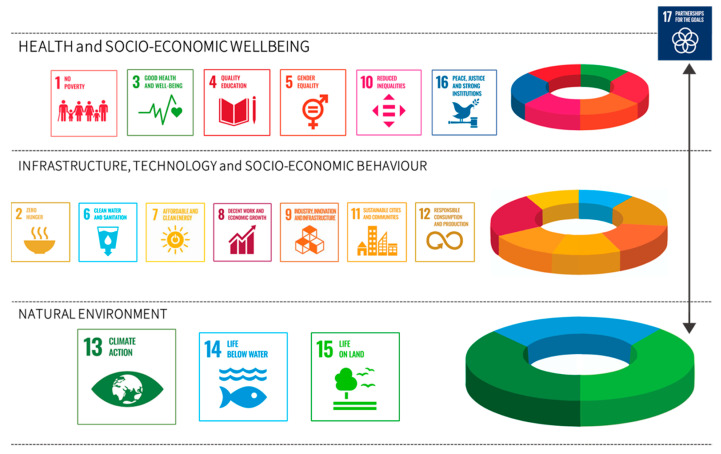
A Sustainable Development Goals approach to harmonizing the natural environment with anthropogenic structures (reflected in infrastructure, technology and socio-economic behaviour) to support health and wellbeing. This Figure 2 is developed from insights from [21,24] and is not intended to be comprehensive.

Trends in detrimental global climate and other environmental changes have the potential to reverse recent health gains unless policies are implemented to protect and promote sustainable environmental public health. These health gains are a priority, and their sustainability can be ensured in collaboration with all responsible sectors, for instance agriculture, water, urban planning and energy, at local to national and global scales. Policies such as those suggested by the Sustainable Development Goals are good examples of how the health workforce could approach and understand sustainability. Figure 2 suggests how the Sustainable Development Goals might provide a structure for harmonizing the natural environment with anthropogenic constructs (reflected in infrastructure, technology and socio-economic behaviours) that are critical for protecting and promoting goals associated with health and wellbeing [5,21,22]. The health workforce should know, understand, and where applicable, apply and act to halt and reverse any loss of health gains. It should become part of the systems of preventative and developmental policies.

## 3. Health Worker CECH Competence

A climate- and environment-resilient health system is one that is capable to anticipate, respond to, cope with, recover from, and adapt to shocks and stresses caused by climate, natural and anthropogenic environmental processes, so as to bring sustained protection of and improvement in population health, despite an unstable climate and stressed local human environments [5,8,9,13]. CECH-competent health workers are key to ensure climate- and environment-resilient health systems. This is a deeply complex work environment that challenges the setting of clear competency goals. Our framework of proposed core CECH competencies addresses what it means to be a competent health worker–but also associated workers in other sectors, to the extent that:

- Health workers know what it means to be CECH competent;
- Health and other workers understand how diverse and tiered health work is;
- Health workforce is now- and next-generation; and
- Health workforce should understand its clear collaborative place in broader delivery of HESET-related services.

### 3.1. What it Means to Be CECH-Competent Health Workers

**Figure 3:**
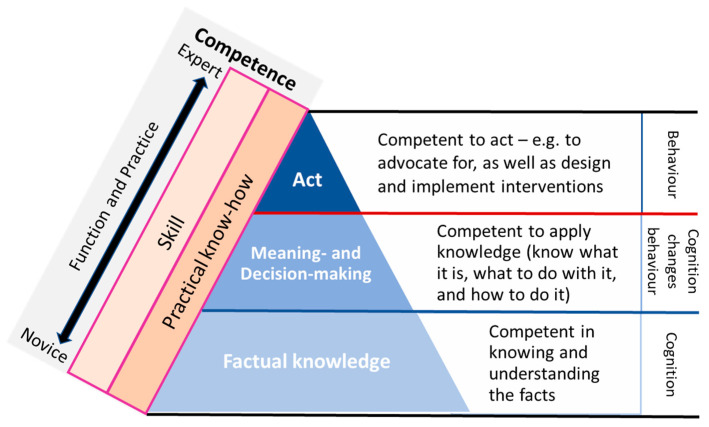
Framework for CECH Health Worker competence. This Health Worker Competency framework is developed from insights from [26,27,28].

Competency occurs when workers gain experience in the world of work-underpinned by early training (e.g., university, college), as well as continual professional development (workplace training)-commensurate with their respective roles, duties and responsibilities [25]. This relationship is reflected in Figure 3.

A novice health worker will complete early career training and enter the workforce perhaps more knowledgeable about the facts (factual knowledge), but less experienced about how to apply and act on these facts. The blue triangle shows the competency progression through learning, which is what the educational/training approach strive to achieve. The orange blocks show that the worker progresses through a career by building practical knowledge and skill on top of their educational foundation. Through continual learning eventually becoming an expert by gaining ‘knowing and understanding real life problems’ (from factual to practical knowledge) as well as ‘what to do about it’ (apply and act–which is the skill). The workforce therefore becomes more competent through function and practice over time-from being newly qualified novice health workers to become experts at what they do.

In terms of undergoing early and continual professional development, the levels of competence a health worker starts off with, and will ultimately achieve, will depend on how they develop their cognitive abilities and executive behaviours. Health workforce education in general show many activities developed to improve individual professional competence according to discipline-based requirements. Initially during early training, study and early career working, the worker progresses from *factualknowledge *to *application* (know what to do). Ultimately this leads to competent professional behaviour demonstrated by the ability to *act* through making and implementing good decisions commensurate with their respective roles, duties and responsibilities.

### 3.2. Health Work Is Diverse and Tiered

The health workforce includes a diversity of health disciplines. Responsibilities of health workers include responding to impact (clinical/curative) and preparing for (population health-related prevention, mitigation, adapting to) the detrimental health risks of climate and environmental change.

Health workers work in different tiers, but all should be required to have some competencies (knowing, applying, and action) of intra-sectoral (e.g., within the health sector, the public health, medical and emergency health services) and health-determining intersectoral services (e.g., environment and social services support for health).

Tiers of health work include:

- Local/community level workers
- Regional and national level decision makers of collective service delivery (preventative and curative)-i.e., local health care centres, country level ministries, and regional level organisations and agencies, such as WHO regional and country offices.
- Global level–agencies and global operators in CECH, including WHO headquarters in Geneva and other UN organizations.

This means that competent health workers should be able to integrate complex CECH knowledge and skills across environment, infrastructure, exposures, cultures, behaviours and health in many different settings from local to global. Depending on the level at which the health worker functions, these integrations will range from lower cognitive levels of ‘knowledge’ (knowing about things) to the increasingly challenging making executive decisions at ‘higher’ levels (apply and act).

The health workforce is increasingly required to make decisions on CECH within the level (tier) of their practice/application and, as is often the case where they are the highest qualified professionals around, also support decision making at levels higher than their own. It is critical that their CECH competencies match this requirement.

### 3.3. Health Workforce Is Now and Next Generation

As the next generation of health workers (i.e., university leavers, early career professionals) progress their competence from novice to expert (decision-making) levels, they should develop their competencies throughout to efficiently address and manage CECH issues, even as these issues become more complex with additional climate and environmental change (stay in touch). Gaps in competencies can be addressed by employers and professional associations in collaboration with accredited training institutions.

### 3.4. Health-Determining Intersectoral Collaboration

The core competencies we propose are designed to ensure that health decision-makers and operational health workers have the base knowledge and skills to work effectively with all health-determining sectors of society to identify, prioritize, implement, and monitor new and modified policies and programs to build resilience to a changing climate and environment. This includes working with the organizations and agencies that manage water and sanitation, food and agriculture, energy, urban planning, and more.

Health system infrastructure and functioning also are affected by climate and environmental change. Health workers, in collaboration with engineers and other specialists, should have competencies to assess the extent to which services and infrastructure could be susceptible to extreme events and to plan for how to prepare for and manage events more extreme than historically experienced.

Health service providers should train and support professionals and staff in these other sectors to ensure that their prevention and response strategies (environment, social, economics, technological) do not inadvertently harm population health, but protect and promote health.

## 4. Core Competency Domains and Sub-competencies for Climate and Environmental Change and Health

This is what the Core CECH competencies are proposed to achieve. We do not provide the detailed content of how the health workforce should be trained across the competence framework in Figure 3. Instead, we provide six core competencies from which detailed training modules could be developed across the Competency Framework (Figure 3), as local, regional and global needs require. To facilitate ever-growing needs for CECH-competent health workers, training institutions and continuing professional development providers (within workplace, professional body memberships, health and other ministries and other organizations, i.e., WHO) could apply these competencies to broaden the factual knowledge, application and acting competence while further achieving and integrating practical knowledge and skills.

These proposed competencies are different from typical domains of health professional competencies [29] in that they are aimed at supporting contextually ‘hard’ CECH competencies. The framework shown in Figure 3 above reflects this paradigm for building broad CECH competence in health workers.

Core structuring of CECH competencies is challenging considering the myriad of climate and environmental issues that can affect health. Attaining the necessary levels in quantity, quality, and relevancy in the health workforce will require clear alignment on these competencies between the education sector and health labour market to meet evolving needs [11].

The competencies in Table 1 are therefore aligned with the CECH needs of the population and the health workforce that serves them and practices within them. Using this framework for curricula development will require assessment of these competencies within the scope of practice of CECH. It will be for training institutions to develop and assess whether the desired level of competencies where achieved-in collaboration with the various sectors involved in CECH [11]. Progress towards achieving competence will thus be defined by the quality and relevance of the knowledge (factual as well as practical) and skills of new graduates as well as those undergoing continual professional development over the course of their careers.

To facilitate competency development, core competencies are clustered to reflect ‘domains’ of CECH competence with sub-competencies that more closely reflect the required knowledge and skills. Table 1 lists the core competencies in 6 domains.

The CECH competency framework shown in Table 1 provides the domain-based blueprint for the health sector as well as universities and other training organizations to develop these competencies in health workers from local to international levels. Sub-competencies for each domain are shown Table 2, Table 3, Table 4, Table 5, Table 6 and Table 7 in the sub-sections to follow.

The competency sets are intended to be flexible to allow for institutions such as universities, professional bodies, service providers and other implementing agencies to collaborate and further refine the competency attributes that their novice as well as more experienced learners should develop. The particular health discipline, as well as tiers of work, will determine to what extent a health worker should be trained to ‘know, apply, and take action’ according to the competency domains (Table 1.) and their under-builds (Table 2, Table 3, Table 4, Table 5, Table 6 and Table 7 in the following subsections).

**Table 1:** Core Climate and Environmental Change for Health Competencies for education and training of Health Workers.

DOMAIN	CORE COMPETENCY
**1**	Climate, Environmental Change and associated Health Sciences
**2**	Upstream Drivers of Climate and other Environmental changes
**3**	Evidence, projections and assessments
**4**	Iterative risk management
**5**	Mitigation, adaptation and health co-benefits
**6**	Collective strategies–harnessing international/regional/local agreements and frameworks

### 4.1. Domain 1: Climate, Environmental Change and Associated Health Sciences

Competence in this domain is predominantly about factual knowledge (know facts and understand them) of climate, environment and related health sciences. Using this knowledge would be commensurate with the tier at which the health worker functions–for instance a local/community health worker might want to train community workers in general CECH sciences. The CECH sciences include natural and anthropogenic changes in the environment–for instance know and understand aspects such as mean and variability of weather variables (e.g., temperature and precipitation), the environmental changes they cause (e.g., flooding) and associated health outcomes given exposure (where communities live) (Table 2).

Considering that this knowledge extends through application (making decisions) to action, Domain 1 is conceivably the leading domain because without sufficient and appropriate factual knowledge about the science of CECH, health workers will end up being less competent or confident to apply (decide on the course of action) and act (gather evidence, plan, manage risks) meaningfully.

**Table 2:** Competencies for Domain 1: Climate, Environmental Change and associated Health Sciences.

Context	Climate and environmental change alter current and future geographic ranges, seasonality, and consequently associated health outcomes
	Healthy planet for healthy people
Climate change	Weather, climate, climate variability and relationships with other environments
Environmental change	Natural and anthropogenic changes, including relationships with climate
Hazard and exposure	Hazard and exposure pathways
Health outcomes	Health outcomes of specific climate and environmental changes

### 4.2. Domain 2: Upstream Drivers of Climate and other Environmental Changes

These drivers interact with climate and environmental changes to determine the magnitude and pattern of risks. These drivers include those from populations pursuing the basic necessities of life (food and shelter) and beyond. These in turn create pressures on the environment such as depletions of natural resources and wastes from excessive consumption (Table 3).

**Table 3:** Sub-competencies for Domain 2: Upstream Drivers of Climate and other Environmental changes.

Drivers	Population, distribution, and needs
	Disparities and aspirations
	Economic development
	Technology
	Energy needs
Pressures	Depletion of natural resources
	Waste and pollution
	Shifts in natural balancing processes–i.e., excessive infectious disease outbreaks

### 4.3. Domain 3: Evidence, Projections and Assessments

In many instances, Domains 1 and 2 competencies are transitional between meaning- and decision-making competencies (application) and action competencies of Domains 3–5.

A health worker should be competent–at the level commensurate with the tier they are working at–to gather evidence through research, tracking, monitoring, surveillance. A competent health worker should then be able to assess–from the evidence-the many states/conditions of CECH at their levels of practice. Where data may be limited or absent, a competent health worker should be able to generate expert judgements and/or understand the evidence and judgements of other CECH scientists and practitioners across broader areas of health, environment, society, economy and technology (HESET) (Table 4).

**Table 4:** Sub-competencies for Domain 3: Evidence, projections and assessments.

Evidence	Surveillance, monitoring, early warning and evaluation systems and plans
	Regional information systems and Centres of Excellence to conduct assessments, data analyses, research and implement actions.
	Detection and attribution of current and past impacts of climate change on health
Project risks	Predictive modelling of the harms and benefits of climate and environmental change for physical and mental health, taking other drivers into account
Research and exploration	Understand current and future climate and environmental changes and their associated health and wellbeing outcomes
Assessment	Methods and capability to conduct vulnerability, capacity, and adaptation assessments
	Baseline assessments of the effectiveness of policies and programs
	Integrating environment and health in local and national information systems

### 4.4. Domain 4: Iterative Risk Management

Iterative or adaptive risk management of CECH refers to being competent at integrating the competencies from Domains 1,2 and 3, to improve CECH decision-making and develop future risk management strategies. This involves incorporation of the findings from assessments into plans for dealing with possible adverse consequences and especially advocating, as well as actively engaging the community, collaborative sectors, and other higher-level stakeholders in these plans (Table 5). Health workers should collaborate closely with those in other HESET services and practices to plan responses accordingly and have the appropriate ability to qualitatively or quantitatively project possible risk based on the evidence, projections and assessments.

**Table 5:** Sub-competencies for Domain 4: Iterative risk management.

Partnership	All sectors involved in environmental protection
	All sectors involved in environmental health services delivery
Vulnerability	Population groups and regions that are particularly vulnerable to hazards of climate and environmental changes
Resilience	Resilient and sustainable infrastructure and technologies
	Resilient environmental health services
Planning	For climate-related shocks and stresses and other environmental changes (i.e., disaster management)
Policy and practice	Integrate evidence and projections into ongoing and future adaptation and mitigation policies and programs
	National response planning processes, including nationally determined contributions, national adaptation plans, and Sustainable Development Goals
	Community consultation/participation in interpretation, integrating evidence into decision-making processes and action
	Monitoring, evaluation and learning of policies and program to manage health outcomes of climate and environmental change

### 4.5. Domain 5: Mitigation, Adaptation and Health Co-Benefits

Health workers must be competent in advocating actions (based on knowledge and understanding) that could be taken to reduce environmental impact especially from their own activities. For instance, greenhouse gas emissions from healthcare and public health infrastructure, particularly energy efficiency and use of renewables. Doing so would encourage and support other sectors to follow (Table 6).

**Table 6:** Sub-competencies for Domain 5: Mitigation, adaptation and health co-benefits.

Health sector	Contributions of health systems to hazardous emissions and other wastes
Community	Options to reduce emissions and wastes including community and other stakeholder participation
Benefits	Health benefits of reducing depletions, emissions and wastes
Co-benefits	Health co-benefits of contributions by other sectors, services and society-driven actions, such as circular economies

### 4.6. Domain 6: Partnerships and Collective Strategies-International and Regional, Agreements and Frameworks

Health workers should be competent to access, engage and use aid and support processes, and know where and when to apply them but not only at the higher tiers –the local/community tiered workers too should at least be aware of these if not knowledgeable–especially in the context of the rapid changes in our environment and climate (Table 7).

**Table 7:** Sub-competencies for Domain 6: Partnerships and collective strategies-international and regional, agreements and frameworks.

Partnerships	Sustainable Development Goals
	Locally, regional and global communication (including advocacy) to include health into climate and environmental change discussions
	Civil society organizations and non-governmental organizations to increase community outreach, raise awareness of participatory mechanisms and engagement in processes and action
	Governance sectors to enhance health-protecting and promoting services
Frameworks	Global and regional frameworks for assessing, managing and reporting on health risks and impacts of climate and environmental change, e.g., the United Nations Framework Convention on Climate Change (UNFCCC), including the Paris and subsequent agreements, and Sendai Framework for Disaster Risk Management

## 5. Discussion

To support and promote environmental public health and reduce risks, health workers should be sufficiently competent to effectively fulfil their respective roles and responsibilities towards managing health-related risks and impacts of climate and other environmental changes. They should have sufficient knowledge and understanding of the state of the global as well as local climate and environment, including how these are changing, and the major drivers and other factors that influence the changes. They also need to understand the pathways, critical events, and opportunities, protective and detrimental, that may lead to people’s health and health systems being affected by-as well as affecting–our climate and environment. This includes food and water security. In addition to health, people’s economic and social wellbeing are being affected [5].

Armed with such knowledge and understanding, health workers will develop insights into the evidence of current and potential future consequences of CECH. They will come to understand and communicate the benefits of reducing these risks and impacts, be able to participate in assessments of risk and impact and provide input into plans for averting these through iterative risk management [30]. To do this effectively, they need to know the main responses and policy measures that should be in place to strengthen environmental and public health protection and management across all sectors of government. They should be sufficiently competent to support discourses and decisions on the effectiveness of management practices in terms of improving environmental and public health quality and resource use [5]. They should know how to advocate across sectors for the health co-benefits of effective decisions about environmental benefits, responsibilities, and risks [17].

Depending on the level (tier) of practice, health workers should be aware, and–where required–be able to consider, develop, implement, and monitor mitigation and adaptation policies, including multilateral Environmental and Health Agreements. These policies also should support achieving the Sustainable Development Goals and other international and national agreements (see Figure 2), to transform the global as well as local climate-environment-health nexus to a more sustainable, and healthy planet for all [5,13,22].

## 6. Conclusions

This framework for core climate and environmental change competencies for education and training of health workers was designed to be useful and flexible for educational programs in health and medical organizations and institutions. The relevance and comprehensiveness of this framework will be tested as it is applied, and the core competencies refined. This means that employers and academic institutions should collaborate with the professional bodies and the service providers on a harmonised approach to training, developing the attributes (training institutional goals) and competencies required for the workplace. This should include processes to collect feedback and recommendations for updates. Doing so would help maintain the relevance of this CECH Core Competency Framework and increase the competency levels of health workers to prepare for and manage the magnitude and pattern of health risks from continuing global environment changes.

## Data Availability

Not applicable.
